# Sonographic signs and patterns of COVID-19 pneumonia

**DOI:** 10.1186/s13089-020-00171-w

**Published:** 2020-04-21

**Authors:** Giovanni Volpicelli, Luna Gargani

**Affiliations:** 1 Emergency Medicine, San Luigi Gonzaga University Hospital, Turin, Italy; 2 Institute of Clinical Physiology, National Research Council, Pisa, Italy

## Abstract

The pandemic of COVID-19 is seriously challenging the medical organization in many parts of the world. This novel corona virus SARS-CoV-2 has a specific tropism for the low respiratory airways, but causes severe pneumonia in a low percentage of patients. However, the rapid spread of the infection during this pandemic is causing the need to hospitalize a high number of patients. Pneumonia in COVID-19 has peculiar features and can be studied by lung ultrasound in the early approach to suspected patients. The sonographic signs are non-specific when considered alone, but observation of some aspects of vertical artifacts can enhance the diagnostic power of the ultrasound examination. Also, the combination of sonographic signs in patterns and their correlation with blood exams in different phenotypes of the disease may allow for a reliable characterization and be of help in triaging and admitting patients.

## Introduction

Chest imaging is in the front door in the diagnostic approach to any patient with respiratory and infective symptoms during this COVID-19 outbreak. The novel corona virus has a specific tropism for the low respiratory airways and the main complication of the disease is pneumonia. Analysis of chest CT scans from patients with COVID-19 allowed important conclusions about the main aspects of the disease [1–3]. The characteristic feature visible in all patients with pneumonia is the ground glass opacification (GGO), a descriptive term indicating interstitial alteration of the lung parenchyma. This was variably associated with peripheral consolidations and crazy paving, a more advanced interstitial alteration where the GGO is combined with interlobular thickening. COVID-19 typically induces an interstitial diffuse bilateral pneumonia with lesions in asymmetric and patchy distribution involving mainly the lung periphery, which makes it particularly suitable for an ultrasound investigation. Alternation of GGO with crazy paving and consolidations can be well depicted by lung ultrasound (LUS). Finally, LUS imaging is also useful to observe the regional distribution of these patterns and describe the patchy bilateral spread of lesions.

## Main signs at lung ultrasound

The sonographic signs of interest in COVID-19 include all those which are well known in ARDS. These are the B-lines in various forms, both separate and coalescent, irregular or fragmented aspect of the pleural line, and small peripheral consolidations. Explanations and demonstrations of all these signs can be easily found in the vast existing literature on the topic [4]. However, in the diagnosis of COVID-19 some specificities need to be considered.

*B*-*lines* B-lines in COVID-19 pneumonia are visualized in all their possible forms. We may describe COVID-19 pneumonia as a storm of clusters of B-lines, both in separate and coalescent forms, sometimes giving the appearance of a shining white lung. They can arise from one point of the pleural line and from small peripheral consolidations and spread down like rays maintaining their brightness until the edge of the screen without fading. These artifacts represent the typical signs of the disease, but can be also observed in other interstitial diseases of various etiologies [4]. However, we are observing that one peculiar aspect of these artifacts is invariably visualized in the early phases of COVID-19 pneumonia (unpublished data). It is a shining band-form artifact spreading down from a large portion of a regular pleural line, often appearing and disappearing with an on–off effect in the context of a normal A-lines lung pattern visible on the background (Additional file 1: Video S1, Additional file 2: Video S2, Additional file 3: Video S3, Additional file 4: Video S4, and Additional file 5: Video S5). In our opinion, this sign is demonstrative of a very acute phase of GGO lesions during the early spread of the active disease, when limited areas of lesions alternate with preserved lung parenchyma. Other Chinese authors called this sign “waterfall”, without further characterizing it [5]. They did not differentiate this vertical artifact from other less specific signs, like coalescent B-lines arising from peripheral consolidations or from a very irregular pleural line. We think that the name “light beam” can well describe this artifact, as a *large beam of light* sometimes appearing and disappearing during respiration. Identifying this band-form sign as the one arising from a large portion of a regular pleural line helps characterizing the LUS pattern. As a technical note, it is crucial to use a convex probe with a large emission surface and low frequency to visualize the light beam more reliably. It is also important to position the focus at the level of the pleural line to prevent misinterpretations of the vertical artifacts.

*LUS patterns* All the LUS signs of COVID-19 pneumonia, including the light beam, can be observed in a variety of different lung conditions. However, what gives specificity to LUS is the distribution of the pattern and the current epidemiological milieu. Bilateral patchy distribution of multiform clusters, where all these signs are represented and sharply alternated to “spared areas”, is typical of the disease. Included in the clusters, evidence of the light beam is crucial to assign a diagnosis of *high probability*. Any other combination of signs should be considered at *intermediate probability* and should demand further testing. Finally, some patterns allow ruling out the disease and orientating towards *alternative* diagnoses. For instance, a regular pleural line with more uniform, symmetric and gravity-related distribution of B-lines with a stronger correlation to the severity of dyspnea, is typical of cardiogenic pulmonary edema. Diffuse irregularities of the pleural line without the typical patchy distribution are more typical of chronic diffuse interstitial pulmonary diseases, like fibrosis. Isolated large lobar consolidation with or without effusion and with dynamic air bronchograms indicates bacterial infection. Large pleural effusion with atelectatic consolidation of the base of the lung and signs of peripheral recruitment during inspiration suggest a compressive origin of the lung condition. The presence of echoic septa or other images inside the effusion demonstrates a different origin of the infection, as SARS-CoV-2 does not yields complex exudative effusions.

*Correlation with the patient condition* The power of LUS in orientating the management of patients during this COVID-19 outbreak is consistently increased by correlating LUS patterns with clinical information. The early approach should be differentiated in three main subgroups of patients, or phenotypes. (1) Patients with respiratory symptoms, ranging from those complaining of mild exertional dyspnea to those with severe respiratory distress; it should be noted that patients with COVID-19 tend to appear less symptomatic than expected when gas analysis or quick exercise testing are performed; (2) patients without respiratory symptoms, suspected for a mild form of SARS-CoV-2 infection; (3) patients with pre-existing chronic cardio-pulmonary diseases, mainly severe COPD, pulmonary fibrosis, lung cancer, cor pulmonale, heart failure.

Three main principles justify this differentiation. (A) A negative LUS examination in patients of the phenotype 1 allows ruling out COVID-19 pneumonia with very high sensitivity and reallocate the patient; sometimes, even an intermediate pattern that is disproportionate to the severity of a respiratory distress may be useful to orientate to another diagnosis; anyway, LUS can exclude the diagnosis of pneumonia but cannot exclude SARS-CoV-2 infection; (B) in phenotype 2, even intermediate LUS signs allow diagnosis of COVID-19 pneumonia that needs to be confirmed by viral swab testing; the degree and distribution of the typical patterns in combination with clinical information allow establishing the severity of the disease and the risks of discharging home the patient; (C) in phenotype 3, any LUS pattern that cannot be considered at high probability, for instance due to absence of multiple light beams, remains doubtful. Diagnosis cannot be concluded without a combination of swab test and often CT scan.

Finally, correlation with timing of the onset of symptoms should be always considered. When the LUS pattern is the result of several days of disease, it is potentially less evolutive than similar patterns observed at a very early phase. The correlation of the LUS pattern with some blood exams is also useful. The typical blood assays picture in COVID-19 is based on the evaluation of leukocyte count, lactate dehydrogenase, procalcitonin, and others. The leukocyte count, which is almost invariably reduced in COVID-19, is especially helpful. Negative serum procalcitonin allows to support the diagnosis of COVID-19 in patients showing LUS signs of pneumonia [6].

## Conclusion

Implementation of LUS during the COVID-19 outbreak is of great interest. LUS allows identification of early signs of interstitial pneumonia. Patterns with combination of different signs allow differentiating positive high probability from intermediate probability of the disease and indicate alternative diagnoses. The correlation of different LUS patterns with the clinical condition at presentation, timing of symptoms onset and a few blood exams, allow a better characterization of the disease at presentation in the ED.

## Supplementary information

**Additional file 1: Video S1.** Lung ultrasound scan from a patient with confirmed COVID-19. The video clip was recorded on the right anterior chest. The clip shows the “light beam” sign with on–off effect.

**Additional file 2: Video S2.** Lung ultrasound scan from a patient with confirmed COVID-19. The video clip was recorded on the left lateral chest. The clip shows the “light beam” sign with on–off effect.

**Additional file 3: Video S3**. Lung ultrasound scan from a patient with confirmed COVID-19. The video clip was recorded on the right superior lateral chest. The “light beam” sign is clearly shown, alternating with multiple separated B-lines and A-lines.

**Additional file 4: Video S4.** Lung ultrasound scan from a patient with confirmed COVID-19. The video clip was recorded in the left superior lateral chest. The “light beam” sign is clearly shown, alternating with multiple separated B-lines and A-lines.

**Additional file 5: Video S5.** Lung ultrasound scan from a patient with confirmed COVID-19. The scan was performed in the left anterior chest. Two areas with “light beam” sign are clearly visible. On the left side the artifact is also combined with an irregular pleural line. When the “light beam” arises from a regular pleural line, as on the right side of the scan, the sign is more specific of the early phase of COVID-19 pneumonia.

## Data Availability

Availability of data is not applicable to this article.

## Abbreviations

COVID: Corona virus disease
SARS-CoV-2: Severe acute respiratory syndrome corona virus-2
CT: Computerized tomography
GGO: Ground glass opacification
LUS: Lung ultrasound
ARDS: Acute respiratory distress syndrome
COPD: Chronic obstructive pulmonary disease
ED: Emergency department
